# A Pilot Study Protocol for AI-Assisted Interpretation of Chest X-rays for Pulmonary Abnormalities in Uganda

**DOI:** 10.7759/cureus.110504

**Published:** 2026-06-09

**Authors:** Johnes Obungoloch, Julius Tumusiime, Jacob Nkwanga, Mugyenyi R Godfrey, Chrispus Mbusa, Fred Kaggwa, Leo Anthony Celi, Jessica E Haberer, William Wasswa

**Affiliations:** 1 Biomedical Engineering, Faculty of Applied Sciences and Technology, Mbarara University of Science and Technology, Mbarara, UGA; 2 Biomedical Engineering, Mbarara University of Science and Technology, Mbarara, UGA; 3 Internal Medicine, Faculty of Medicine, Kabale University, Kabale, UGA; 4 Obstetrics and Gynecology, Faculty of Medicine, Mbarara University of Science and Technology, Mbarara, UGA; 5 Obstetrics and Gynecology, Divine Mercy Hospital-Father Bash Foundation, Mbarara, UGA; 6 Administration, Mbarara University Data Science Research Hub, Mbarara, UGA; 7 Computer Science, Faculty of Computing and Informatics, Mbarara University of Science and Technology, Mbarara, UGA; 8 Medicine, Beth Israel Deaconess Medical Center, Boston, USA; 9 Internal Medicine, Massachusetts General Hospital, Harvard Medical School, Boston, USA

**Keywords:** artificial intelligence, chest x-ray, pneumonia, pulmonary complications, study protocol

## Abstract

Background: Timely access to chest X-ray (CXR) imaging and interpretation remains a practical challenge in routine pulmonary care in Uganda, particularly in settings with limited specialist availability and diagnostic capacity. This study aims to develop a structured, locally derived dataset of annotated CXR images linked with clinical metadata and to evaluate the feasibility of a machine learning model to support the diagnosis of pulmonary conditions. The algorithms developed using metadata acquired in this study will also help predict which patients should be referred for chest X-ray imaging.

Methods: This pilot cross-sectional study will enroll 420 participants from Mbarara Regional Referral Hospital and Divine Mercy Hospital. Consecutive sampling will be used to recruit patients undergoing CXR for suspected pulmonary conditions, as well as individuals with normal findings. De-identified CXR images will be linked to standardized clinical metadata, including demographics, symptoms, examination findings, and imaging parameters. Images will be labeled by trained clinicians using standardized protocols. The dataset will be partitioned into training, validation, and test sets. Machine learning models, including convolutional neural networks and multimodal approaches integrating imaging and metadata, will be developed and evaluated using receiver operating characteristic-area under the curve (ROC-AUC), sensitivity, specificity, precision, recall, and F1-score.

Results: The study is expected to produce a curated dataset of 420 annotated CXR images, including both normal and pathological findings. A pilot machine learning model for identifying pneumonia will be developed and internally validated. Additionally, a regression-based model is anticipated to explore patterns associated with CXR utilization in this clinical setting.

Conclusion: This study will establish a locally derived CXR dataset and assess the feasibility of machine-learning-based diagnostic support in pulmonary care. The findings will inform future model refinement, external validation, and potential integration into clinical workflows in similar settings.

## Introduction

Chest X-ray (CXR) is a cornerstone of pulmonary care, offering a non-invasive means of visualizing thoracic structures and guiding management of conditions such as pneumonia, cardiomegaly, hydrothorax, and trauma-related injuries [1]. Recent advances in medical technology have accelerated the use of AI to augment disease detection and management functions that have historically relied on human expertise [2]. Machine learning (ML), a core subset of AI, enables algorithms to learn from data and generate predictions with minimal manual input⁠, making it well-suited to large-scale analysis of CXR images [3]. ML models can interrogate extensive imaging datasets to identify radiographic patterns such as consolidations, fractures, or effusions with increasing accuracy and efficiency [4]⁠. Patient metadata refers to structured, non-image clinical and contextual information (e.g., demographics, symptoms, vitals, medical history, and acquisition parameters) that complements imaging data and improves model performance through multimodal learning.

While CXR-based AI models have been developed and deployed in high-income settings, evidence from low- and middle-income countries (LMICs) remains scarce [5]⁠. In Uganda, the diagnostic pathway typically involves physician-ordered CXRs, image acquisition by radiographers, and interpretation by radiologists⁠, an approach constrained by workforce shortages and delays [6]. AI-powered decision support could enable earlier identification of pulmonary abnormalities in facilities lacking specialists [7]⁠. Most Ugandans rely on public health facilities with limited imaging capacity; even where CXRs are available, radiologist coverage is scarce. The ratio of specialist healthcare workers, including radiologists, to population in sub-Saharan Africa remains very low, with some estimates placing it at over 1:800,000, compared to the World Health Organization's recommended healthcare worker-to-population ratio of about 4.5:1000 [8,9]. Furthermore, many specialist healthcare workers, such as radiologists, practice in cities, putting rural dwellers at risk of delayed and inaccurate image interpretation [8]. Although AI has demonstrated benefit in other screening contexts within Uganda, its application to pulmonary imaging remains limited. In Uganda, a study has shown that AI can provide high-quality, objective decision-making in cervical cancer screening [10]. AI-powered tools could enable lower-level clinicians to achieve accurate diagnoses.

There is currently little information on efforts to integrate AI into the diagnosis of pulmonary abnormalities in Uganda, yet the burden of pulmonary abnormalities is high [11]⁠. The main objective of this pilot study was to create a database of chest X-ray images showing pulmonary abnormalities and to pilot an ML model to aid in the diagnosis of pneumonia, a disease of pilot interest.

## Materials and methods

Study design

This study will use a cross-sectional, observational design involving human participants to develop a dataset of chest X-ray (CXR) images linked with clinical metadata and to assess the feasibility of a machine learning model for pulmonary diagnosis. Participants will be prospectively recruited during routine care following informed consent. Clinical and imaging data will be collected and de-identified for analysis. No experimental interventions will be introduced, and all procedures will follow standard clinical practice.

Study setting

Divine Mercy Hospital is located in Kamukuzi Division, Mbarara city, which is about 270 km south of the capital city, Kampala. The hospital offers both general and specialized medical services. The hospital serves a population from about 15 districts surrounding Mbarara, but receives patients from a wider area of western Uganda and beyond. According to the hospital director, the hospital has a bed capacity of 100 and receives approximately 100 patients per day (inpatient and outpatient), with over 35,000 outpatient visits annually. The radiology department serves approximately 4500 chest X-ray cases annually.

Mbarara Regional Referral Hospital (MRRH), also known as Mbarara Hospital, on the other hand, is a government-owned healthcare facility located in Mbarara city in the western region of Uganda. The hospital has a bed capacity of 600 and receives about "250,000 outpatient visits and 50,000 in-patient visits annually" [12]. It serves as a referral center for the Ankole sub-region, neighboring districts, and surrounding countries, including the Democratic Republic of Congo, Rwanda, Tanzania, and Burundi. The hospital provides specialized and super-specialized services to a catchment area of 12 districts, including the city of Mbarara, serving a population of about 6.5 million people.

Study population

The study population will consist of adult patients (≥18 years) undergoing chest X-ray imaging as part of routine clinical evaluation for suspected pulmonary conditions, including individuals with normal radiographic findings. Inclusion criteria will include patients with a physician's request for chest X-ray imaging and the ability to provide informed consent. Severely ill patients and those unable to take erect chest X-ray images will be excluded.

Sampling size and procedure

A total of 420 participants will be enrolled in this pilot study. Consecutive sampling will be used to recruit eligible participants presenting to the radiology departments of the study sites. Recruitment will occur in accordance with routine clinical indications for chest X-ray examinations to ensure that the dataset reflects real-world clinical workflows.

Data collection procedures

Interview Data

The interviews will be conducted using an interviewer-administered questionnaire in a custom-developed application. The application will be built in-house by the project team. The application shall include a data collection tool, a user interface dashboard for data management of both metadata and image data, and labeling of the collected images.

The study tool will collect the following metadata: (i) sociodemographic characteristics (tribe, age, occupation, level of education, income, and distance from the nearest health facility, smoking and alcohol use, marital status). (ii) Medical history (HIV status and regimen, diabetes, hypertension, allergies). (iii) Presenting symptoms (cough, difficulty breathing, chest pain, fever, night sweats, weight loss, and easy fatigue). (iv) Examination findings (vitals, Eastern Cooperative Oncology Group {ECOG} score, Glasgow Coma Score, anthropometric measurement, weight, body mass, height, and BMI). (v) Respiratory exam findings (modified medical research council dyspnea score, palpation findings, and auscultation findings).

The Klear chest dashboard: The Klear chest dashboard (Mbarara, Uganda: Mbarara University of Science and Technology) is the data analytics platform. It is used to register users, upload images, archive data from the app, and manage the data management process. The dashboard is used mainly for (a) user management - this includes creating accounts for the app, accessing logs for the users, and tracking data entered by each user, (b) image upload - this shall include uploading images into participants’ folders, (c) data quality control - this includes data review both in longitudinal and report formats, data download, editing, and deletion. This data will be checked with the paper records, (d) labeling - this process will include assigning images to the labelers and handling the data, labeling process, including review and approvals, (e) archiving Data Management and Analysis Core (DMAC). The dashboard will be linked with the DMAC storage infrastructure for data archiving. DMAC is the data management and archiving core, a department responsible for the management of collected data stored on the Mbarara University of Science and Technology servers.

Participant Consent and Enrolment

**Figure 1:**
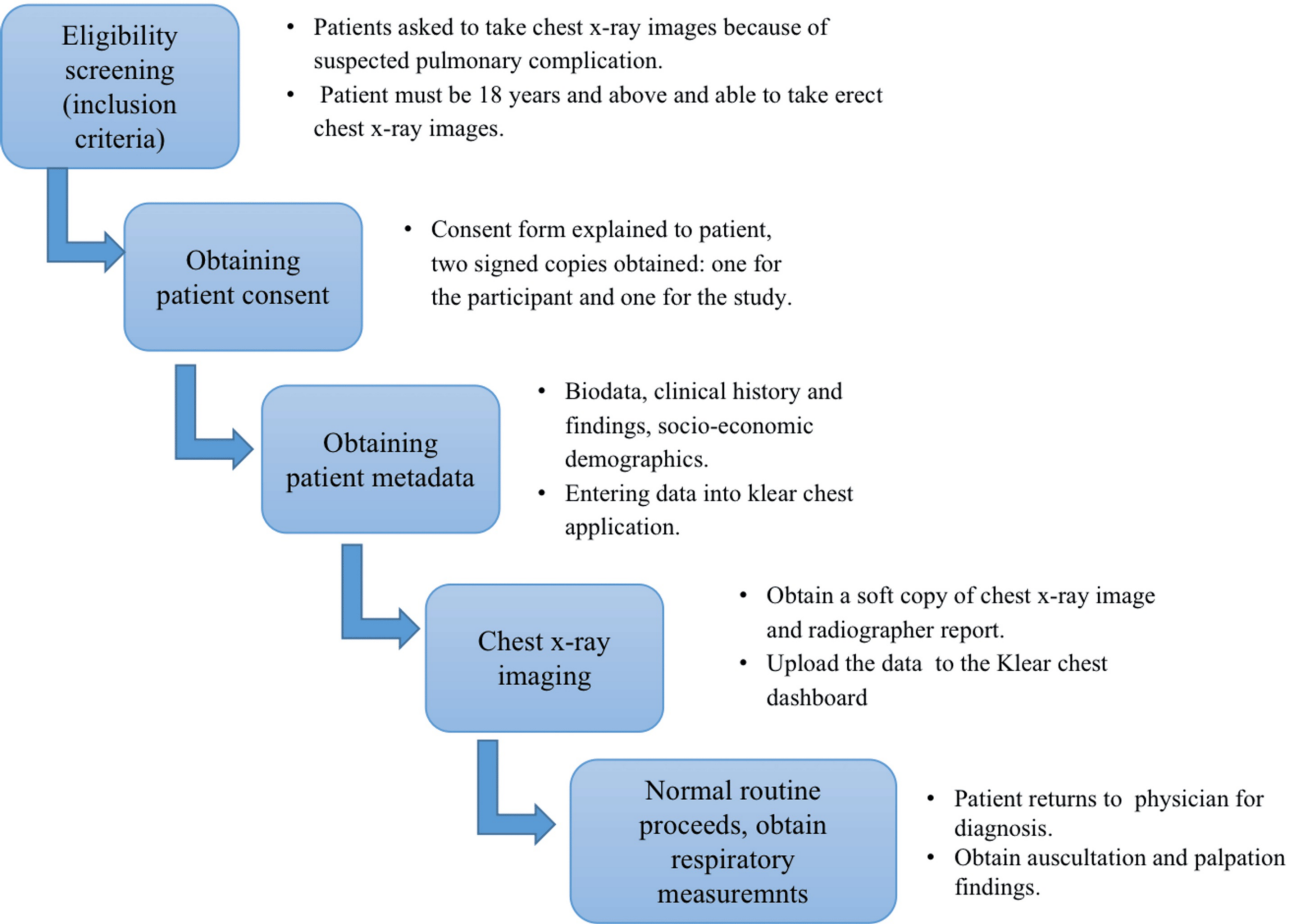
Study procedures and standard operating procedures for participants at enrolment.

The activity will follow specific standard operating procedures (Figure 1 and Table 1). Patients qualifying for our study will be approached by our trained research assistant to seek their consent before recruitment into the study. The consent form shall be explained to the patient in either English or Runyankore, a common language among the people of the region. Written consent shall be obtained, followed by the collection of patient metadata.

**Table 1:** Standard operating procedure for obtaining CXR image using the DRGEM digital X-ray machine. The image is obtained at 70 kVp and 1.2 mAs, as recommended by the DRGEM manufacturers for PA chest exposure; the exposure time is approximately 10 ms. Image is de-identified by removing patient bio data; image is exported in PNG format. The system consists of an X-ray tube assembly: rotating anode X-ray tube, maximum voltage 125 kV, focal spot sizes 1.0/2.0 mm, inherent filtration 1.0 mm Al at 75 kV; a collimator: model DXC-RML (2021 model) (DRGEM: Gwangmyeong-si, South Korea), inherent filtration 2.0 mm Al eq; line power rating 12–15 V DC/20–30 V AC, 50/60 Hz, 42 VA, duty cycle 50%; and a wireless digital flat-panel detector, model Mano4336W. SOP: standard operating procedure(s); CXR: chest X-ray; PA: posteroanterior

SOP/activity	Purpose	Procedure/description	Results	Comment
Equipment preparation	Making the X-ray machine ready and safe for use	Powering the digital DRGEM CXR machine (DRGEM: Gwangmyeong-si, South Korea), entering patient biodata in the DRGEM software (DRGEM: Gwangmyeong-si, South Korea), and powering the detector and ensuring correct placement in the vertical bucky	Ready machine for CXR examination	The machine is fully set up and operated on a camera and microphone communication module between the patient and the radiographer
Patient preparation	Patient readiness for the examination	Remove any metallic material from the region of exposure, that is, the thoracic region. Providing protective gear to patients in other regions, such as the gonads. Position the patient in the PA position on the vertical bucky. Arms wrapped around the vertical bucky	Patient positioned for X-ray imaging	A new workspace is opened in the DRGEM software, the collimator is aligned, and the exposure parameters are set
Image acquisition	Obtaining the CXR image	The X-ray room is closed, the tube is prepared, the patient is asked to take a deep breath over a speaker in the room, and a camera is used for monitoring. Image obtained on the monitor	CXR image	The image is edited and provided as a soft copy in PNG format to the research assistant. The radiographer's report is attached as well

Image acquisition

Equipment

Chest X-ray images will be obtained using the GXR SD series X-ray system (Gwangmyeong, South Korea: DRGEM). This is described as an advanced radiography system that delivers high-resolution images.

Examination

**Figure 2:**
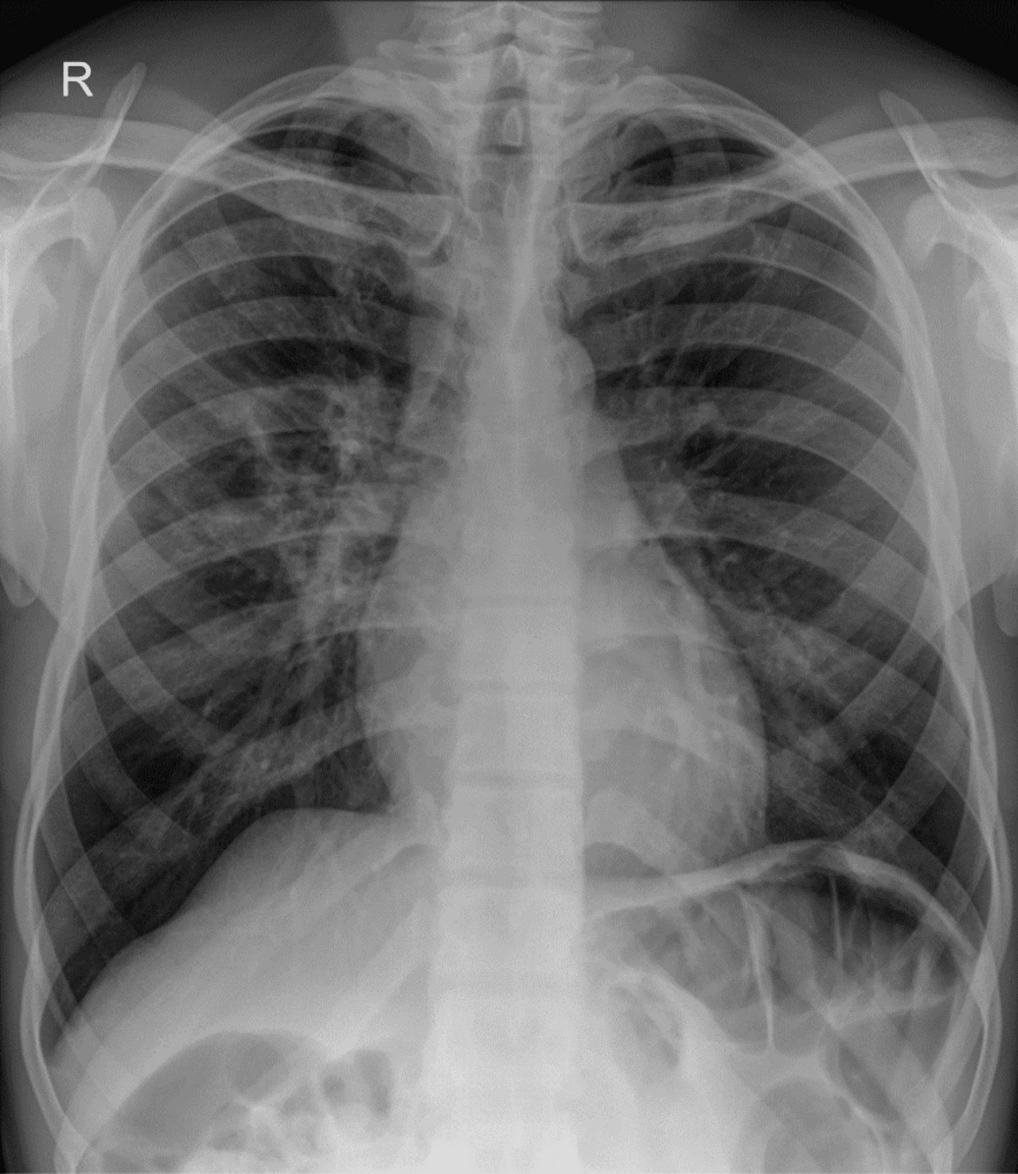
Example of a PA chest X-ray image with de-identified patient data. PA: posteroanterior

The chest X-ray examinations shall be performed to generate images relevant to clinical diagnosis. From each patient, a single erect posteroanterior (PA) image shall be acquired in the study following the procedures shown in Figure 2. The imaging shall be carried out by qualified and trained radiographers for the intended purpose, following the normal standard operating procedures and standardized protocols for performing chest X-ray imaging. Chest X-ray image and radiographer report shall be obtained by the research assistant on a flash disk as PNG files and uploaded to the Klear chest dashboard.

Image Processing and Quality Control

Image processing will be manually done to clean images and enhance quality, including the elimination of extremely blurry and/or low-resolution images. Noise reduction will be done by adaptive filtering, which helps in preserving edges and structural details, and the wavelet transform, which decomposes the images into different frequency components, enabling noise to be isolated and removed without affecting the main image features [13]⁠. For artifact removal, segmentation techniques will be used to identify and isolate regions of interest to separate true anatomical structures from artifacts [14]⁠. For image enhancement, histogram equalization to improve image contrast and adaptive contrast enhancement, which modifies the brightness of different regions, will be used [15]. Image normalization and standardization will involve pixel-intensity normalization, which adjusts the range of pixel intensities; resolution standardization, which resamples all images to a consistent resolution; and orientation correction, which ensures that all images have a consistent orientation [16].

Image Labeling

Expert radiologists and radiographers will meticulously label key anatomical structures and potential abnormalities observed in images related to pneumonia, cardiomegaly, normal findings, and hydrothorax. This will follow a structured, step-by-step image quality assessment (Table 2). Before starting the labeling process, a training session will be conducted for all labelers, who shall include expert radiologists and radiographers. Each image will be independently and blindly labeled by two radiology experts; labels shall be compared for consensus. In the event of discrepancies, a council of senior radiologists with at least 10 years of experience shall jointly review and label the images. Images with inconclusive findings will be excluded from the study. A comprehensive labeling guide will be provided, regular quality checks will be performed, and feedback will be collected from annotators. All files shall be stored in PNG format, in a structured dataset for AI training.

**Table 2:** Steps in assessing image quality. Any image with a "no" response to any criterion shall be excluded from the dataset. PA: posteroanterior

Projection correctly indicated	Yes	No
Patient erect if labeled PA		
Clavicles are symmetric relative to spinous processes (no rotation)		
Medial clavicles are equidistant from vertebral spinous processes		
Scapulae projected outside the lung fields (PA erect)		
Arms properly positioned		
At least 9-10 posterior ribs visible (adequate inspiration for PA)		
Lung fields are adequately expanded		
Vertebral bodies just visible through cardiac shadow (adequate penetration)		
Intervertebral disc spaces are faintly visible		
Lung markings are visible in the peripheral lung fields		
No underexposure (image not excessively white)		
No overexposure (image not excessively dark)		
Both lung apices included		
Both costophrenic angles are visible		
Lateral chest wall margins fully included		
No excessive unnecessary exposure outside the thoracic region		
Sharp rib margins (no motion blur)		
No respiratory or patient motion artifact		
Sharp diaphragmatic outline		
No clothing artifacts		
No metallic artifacts (necklaces, bras, wires)		
No ECG leads or external devices obscuring lung fields		
No grid artifacts visible		
Trachea centrally positioned (unless deviated by pathology)		
No obvious lateral tilt or positioning asymmetry		

Steps in image quality assessment

Data Management and Storage

All collected data, including images and associated metadata, will be de-identified prior to storage. Data will be securely stored in a structured repository hosted by the Data Management and Analysis Core (DMAC) at the Mbarara University of Science and Technology Data Science Research Hub. Access to the dataset will be restricted to authorized study personnel, and all data handling procedures will comply with institutional data protection and governance policies.

Machine Learning Model Development and Evaluation

The machine learning model will be developed and evaluated through a five-stage pipeline comprising: (1) data acquisition and preprocessing, including image enhancement (noise reduction, Contrast Limited Adaptive Histogram Equalization {CLAHE}, resolution normalization, orientation correction), clinical metadata standardization, and data augmentation to improve diversity; (2) dataset splitting into training (70%), validation (15%), and test (15%) sets with stratification to preserve case proportions; (3) model architecture, exploring a custom Convolutional Neural Network (CNN), transfer learning with ResNet50, DenseNet121, and EfficientNet-B3, and multimodal fusion of image and clinical data (20); (4) model training using adaptive moment estimation (Adam) and stochastic gradient descent (SGD) optimizers, appropriate loss functions, early stopping, and learning rate scheduling; and (5) model evaluation with metrics such as accuracy, sensitivity, specificity, receiver operating characteristic-area under the curve (ROC-AUC), and F1-score, statistical significance testing, and gradient-weighted class activation mapping (Grad-CAM) visualizations for explainability. Data governance will be overseen by a dedicated committee to ensure quality, mitigate bias, and maintain reproducibility. As a novel pilot study at these clinical sites, we anticipated that not all 420 enrolled participants would strictly adhere to the imaging protocol. Furthermore, repeating X-rays was restricted due to radiation safety guidelines. Consequently, we utilized a subset of 400 high-quality images for model development. Given the pilot nature of this project and the resulting small dataset, these 400 images will be divided into training and testing phases. To validate the machine learning algorithm, AI-generated interpretations will be compared directly against expert-labeled images.

Study outcomes

The primary outcome of this study is a pilotable machine learning model for the automated diagnosis of pneumonia, a case of interest for piloting. The secondary outcome will be a labeled, de-identified dataset of chest X-ray images linked to their respective metadata.

Ethics and dissemination

Ethical clearance was obtained from the Research Ethics Committee (REC) at Mbarara University of Science and Technology (#MUST-2024-1343), and the pilot equally acquired the national clearance from the Uganda National Council of Science and Technology (UNCST) (#SS2718ES). Study site administrative clearance was obtained from the Hospital Director of Divine Mercy Hospital and the head of the radiology department, Mbarara Regional Referral Hospital. All clinical metadata and images will be de-identified and anonymized before uploading to the DMAC.

## Results

The primary outcome of this pilot project will be the development of an AI tool for predictive diagnosis of pulmonary abnormalities. This will include the development of a machine learning (ML) model to predict pneumonia as a pilot complication. Beyond supporting diagnosis and detection in resource-constrained regions, it is also a metadata regression model for determining which patients are eligible for CXR, with the aim of reducing exposure risk and limiting the number of patients in need of imaging within limited services. Architectures explored will include a custom CNN and transfer learning models such as ResNet50, DenseNet121, and EfficientNet-B3. Fusion of image features with clinical metadata will be evaluated to enhance predictive performance. Training will employ stratified dataset splits (70% training, 15% validation, 15% testing) with augmentation strategies to improve diversity. Performance will be reported using accuracy, sensitivity, specificity, ROC-AUC, and F1-score, with statistical testing and confidence intervals. Explainability will be assessed using Grad-CAM visualizations that highlight clinically relevant regions aligned with expert annotations.

## Discussion

This pilot study is a timely, context-specific effort to harness emerging artificial intelligence techniques to interpret chest X-ray (CXR) images for the diagnosis of pulmonary complications in Uganda. Pulmonary disease remains among the most significant contributors to preventable and treatable mortality in Uganda and East Africa as a whole [17]⁠, largely due to delayed diagnosis, limited imaging capacity, and a shortage of specialist personnel [18]⁠. This study seeks to strengthen diagnostic pathways and ultimately improve pulmonary health outcomes in resource-constrained settings by introducing AI-based diagnosis.

Globally, AI applications in chest radiography have demonstrated strong performance in identifying pneumonia, tuberculosis, trauma-related abnormalities, and other thoracic pathologies [19,20]⁠. However, most of these advances have occurred in high-income settings characterized by robust digital infrastructure, consistent imaging quality, and access to large, well-annotated datasets [21]. In contrast, this study will generate one of the earliest annotated CXR datasets drawn entirely from a sub-Saharan African population. As such, it addresses a critical gap in global AI research, where data from low- and middle-income countries remain significantly underrepresented. Training AI models on locally sourced data enhances clinical relevance, improves generalizability within similar contexts, and advances equity by ensuring that AI tools deployed in Africa are informed by African populations. The inclusion of images from participants with confirmed pulmonary complications and those with normal radiographic findings is a key strength of the study design. This approach enables the construction of a balanced and representative dataset, supporting the development of ML models capable of discriminating between normal and pathological CXR patterns. Moreover, the structured processes for image acquisition, quality control, and expert annotation enhance data reliability and reproducibility. The integration of comprehensive clinical metadata, including medical history, examination findings, social factors, and imaging parameters, provides critical contextual information that supports multimodal model development and validation.

From a broader health systems perspective, this initiative aligns closely with Uganda’s Ministry of Health's digital health strategy and the Sustainable Development Goals (SDGs), which prioritize innovations that strengthen clinical decision-making, expand access to quality care, and reinforce digital health infrastructure [22,23]. Artificial intelligence is increasingly recognized as a transformative tool capable of addressing health workforce shortages and diagnostic inequities, particularly in low-resource environments [24]⁠. In facilities where radiologists and specialist radiographers are scarce, AI-assisted interpretation of medical imaging has the potential to enhance diagnostic accuracy, inform referral decisions, and facilitate timely clinical interventions. These benefits are especially relevant for pneumonia, which continues to be a leading cause of neonatal and under-five mortality in Uganda and across sub-Saharan Africa [25]⁠.

Nevertheless, several limitations of this pilot study must be acknowledged. The relatively modest sample size of 420 participants and images may constrain the generalizability of both the dataset and the resulting machine learning model, the biased data-collection criteria for patient ailment levels, and the small geographical scope of the study. The exclusion of critically ill patients and those unable to stand for PA chest X-ray imaging could have denied the study high-quality images that would inform the better development of algorithms. Model performance will be influenced by consistency in image acquisition and annotation, which may vary due to operator skill, equipment performance, and environmental factors. Additionally, while the project benefits from strong research infrastructure, scaling AI tools across Uganda’s health system would require substantial investment in digital infrastructure, workforce training, and governance mechanisms. Issues related to data protection, informed consent, clinical accountability, and regulatory oversight of AI-generated recommendations will require clear national policy frameworks. The Ministry of Health will play a critical role in establishing standards for validation, deployment, monitoring, and integration of AI tools within existing health information systems. Despite these challenges, this study provides a robust foundation for generating evidence on the feasibility and value of AI-enabled pulmonary diagnostics in resource-limited settings.

## Conclusions

While the study will be conducted at Divine Mercy Hospital and Mbarara Regional Referral Hospital, which are located in a fairly urban area, many of the patients who visit these hospitals, especially for imaging services, come from rural areas, as there are no imaging services in such areas. Therefore, the patient population can be considered a fair representation of Uganda's population. By developing and testing a locally trained machine learning model using CXR images from this study, this study has the potential to address a critical diagnostic gap in the care of pulmonary abnormalities. The findings will not only inform future scale-up and integration of AI into Uganda’s pulmonary care services but also contribute to a growing body of evidence advocating for context-specific AI solutions to global health challenges. This work will demonstrate the feasibility of training a clinically relevant AI model in a low-resource setting using locally sourced and annotated CXR images. By integrating multimodal data (imaging plus clinical parameters), the model is expected to outperform image-only approaches in predictive accuracy.
